# Nanoscale Au-ZnO Heterostructure Developed by Atomic Layer Deposition Towards Amperometric H_2_O_2_ Detection

**DOI:** 10.1186/s11671-020-3273-7

**Published:** 2020-02-17

**Authors:** Hongyan Xu, Zihan Wei, Francis Verpoort, Jie Hu, Serge Zhuiykov

**Affiliations:** 1 School of Materials Science & Engineering, North University of China, Taiyuan, 030051 People’s Republic of China; 2 Department of Green Chemistry & Technology, Ghent University Global Campus, 119 Songdomunhwa-ro, Yeonsu-gu, Incheon, 21985 South Korea; 3 Faculty of Bioscience Engineering, Ghent University, Coupure Links 653, 9000 Ghent, Belgium; 4 State Key Laboratory of Advanced Technology for Materials Synthesis and Processing, Center for Chemical and Material Engineering, Wuhan University of Technology, Wuhan, People’s Republic of China; 5 College of Information Engineering, Taiyuan University of Technology, Taiyuan, 030024 Shanxi People’s Republic of China

**Keywords:** Au-ZnO, Heterostructures, H_2_O_2_, Atomic layer deposition, Amperometric detection

## Abstract

**Abstract:**

Nanoscale Au-ZnO heterostructures were fabricated on 4-in. SiO_2_/Si wafers by the atomic layer deposition (ALD) technique. Developed Au-ZnO heterostructures after post-deposition annealing at 250 °C were tested for amperometric hydrogen peroxide (H_2_O_2_) detection. The surface morphology and nanostructure of Au-ZnO heterostructures were examined by field emission scanning electron microscopy (FE-SEM), Raman spectroscopy, atomic force microscopy (AFM), X-ray photoelectron spectroscopy (XPS), etc. Additionally, the electrochemical behavior of Au-ZnO heterostructures towards H_2_O_2_ sensing under various conditions is assessed by chronoamperometry and electrochemical impedance spectroscopy (EIS). The results showed that ALD-fabricated Au-ZnO heterostructures exhibited one of the highest sensitivities of 0.53 μA μM^−1^ cm^−2^, the widest linear H_2_O_2_ detection range of 1.0 μM–120 mM, a low limit of detection (LOD) of 0.78 μM, excellent selectivity under the normal operation conditions, and great long-term stability. Utilization of the ALD deposition method opens up a unique opportunity for the improvement of the various capabilities of the devices based on Au-ZnO heterostructures for amperometric detection of different chemicals.

**Graphical abstract:**

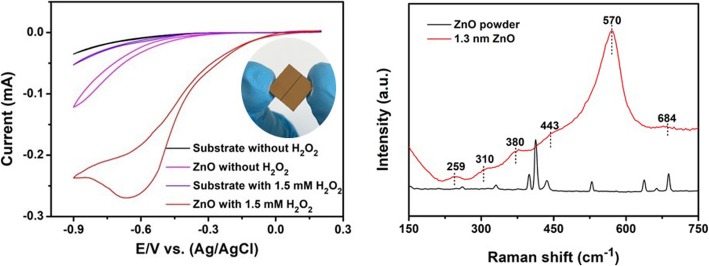

## Background

The development of different nanoscale two-dimensional (2D) heterostructures for their usage in various electrochemical devices is modern and established trend during the last decade of the twenty-first century [1–5]. So far, several technologies have been dominated in this trend including RF sputtering, chemical vapor deposition (CVD), hydrothermal method, solvothermal method, thermal evaporation, sol-gel, mechanical exfoliation, etc. [5, 6]. However, it was clearly stated in the recent review [6] that the ALD as an emerging technology has not yet fully exploited its features towards the development of reliable electrodes for the measuring devices. There are several reasons for that including the availability of precursors, specific ALD temperature window for deposition, lack of reliable recipes, etc. However, on the other hand, the advantages of ALD are far superior to the existing capabilities of other techniques [7]. First of all, ALD is the only one technology, which enables fabrication of *conformal*, defect-free semiconductor 2D nano-films and their heterostructures on the wafer scale with precise control of their thickness during fabrication at the Ångstrom level [8]. In this regard, *state-of-the-art* nanoscale interfacing and molecular engineering of the sensing electrode materials can open up completely new possibilities by providing ultra-thin channels for key doping, minimization of the density of interfacial impurities and optimization of sensing capabilities of the devices [9]. Several recent reports about ALD-developed wafer-scaled monolayers of WO_3_ [8, 10, 11] and TiO_2_ [12, 13] and few-layered MoO_3_ [14, 15], TiO_2_ [16], and their heterojunctions including Au-WO_3_-TiO_2_ [17], Ga_2_O_3_-WO_3_ [18], Au-Ga_2_O_3_-TiO_2_ [19], etc. are recent consecutive evidence of the impact of this technology on the extension of capabilities of materials for electrochemical [14], optical [20], and photovoltaic devices [17].

H_2_O_2_ possesses strong oxidizing and reducing properties and has been extensively used in various applications including different chemical, pharmaceutical and mining, textile, and clinic industries [21]. H_2_O_2_ is also a by-product of enzymatic reactions and its accurate and sensitive detection has been required in food, environmental, and pharmaceutical applications. Consequently, it is critical to detect H_2_O_2_ rapidly and selectively particular at the low concentrations level. So far several methods have already been established for fast H_2_O_2_ detection including colorimetric [22–25], non-enzymatic [26, 27], electrochemical [28–35], chemiluminescent [36–39], and fluorometric [39, 40] measurements. Although all of them demonstrated reasonable results and good repeatability, their main disadvantages include complex separation processes, time-consuming derivation and relatively high running cost. In addition, due to the electro-active nature of H_2_O_2,_ non-enzymatic electrochemical detection method can be considered as a valuable alternative for selective H_2_O_2_ detection [41] considering that it possesses the rapid response with great stability, wide linear detection range, high precision, low cost, and simplicity [2]. In fact, owing to the extremely high *surface-to-volume* ratio in 2D nanomaterials and heterostructures, their employment for such non-enzymatic H_2_O_2_ detection is highly desirable [42]. It was proven that the heterostructured and sandwiched nanomaterials have better analytical sensing performance than the ordinary nanomaterials [43]. On the other hand, their surface functionalization enabled them to increase substantially their sensing capabilities [44] ensuring the development of a combination of two or more dissimilar nano-materials to form the hybrid nanocomposites, especially metal-semiconductor hybrids [45]. Thus, the outstanding properties of Au-ZnO heterostructures for enhancing the analytical sensing performance of electrochemical sensors are due to the following: (i) the Schottky contacted between nanostructured Au and ZnO results in generation of the thicker depletion layer at the Au/ZnO interface compared with the bare ZnO nanostructure; (ii) the inert surface nature of ZnO; and (iii) high selectivity of Au co-catalyst for direct 2e^−^ reaction, which maximizes the H_2_O_2_ detection. For instance, Au-semiconductor hetero-interfaces have a critical effect on the properties of the surface layer particularly if the thickness of such a layer is just a few nanometers. In this regard, ALD technology is imperative for fabrication of the wafer-scaled semiconductors with nanometer thickness. Moreover, ZnO with wurtzite hexagonal structure has been considered as a very promising candidate for the development of such hetero-interfaces. It has a very wide (~ 3.37 eV) bandgap and can be fabricated defect free by ALD [46]. So far, nanoscale ZnO has clearly demonstrated unique properties as well as excellent electrochemical capabilities in photovoltaics, catalysts, batteries, and different chemical sensors [47–53]. However, despite outstanding reported properties and wide usage of ZnO in many applications, Au-ZnO heterostructures have not yet been reported for electrochemical H_2_O_2_ sensing.

**Fig. 1:**
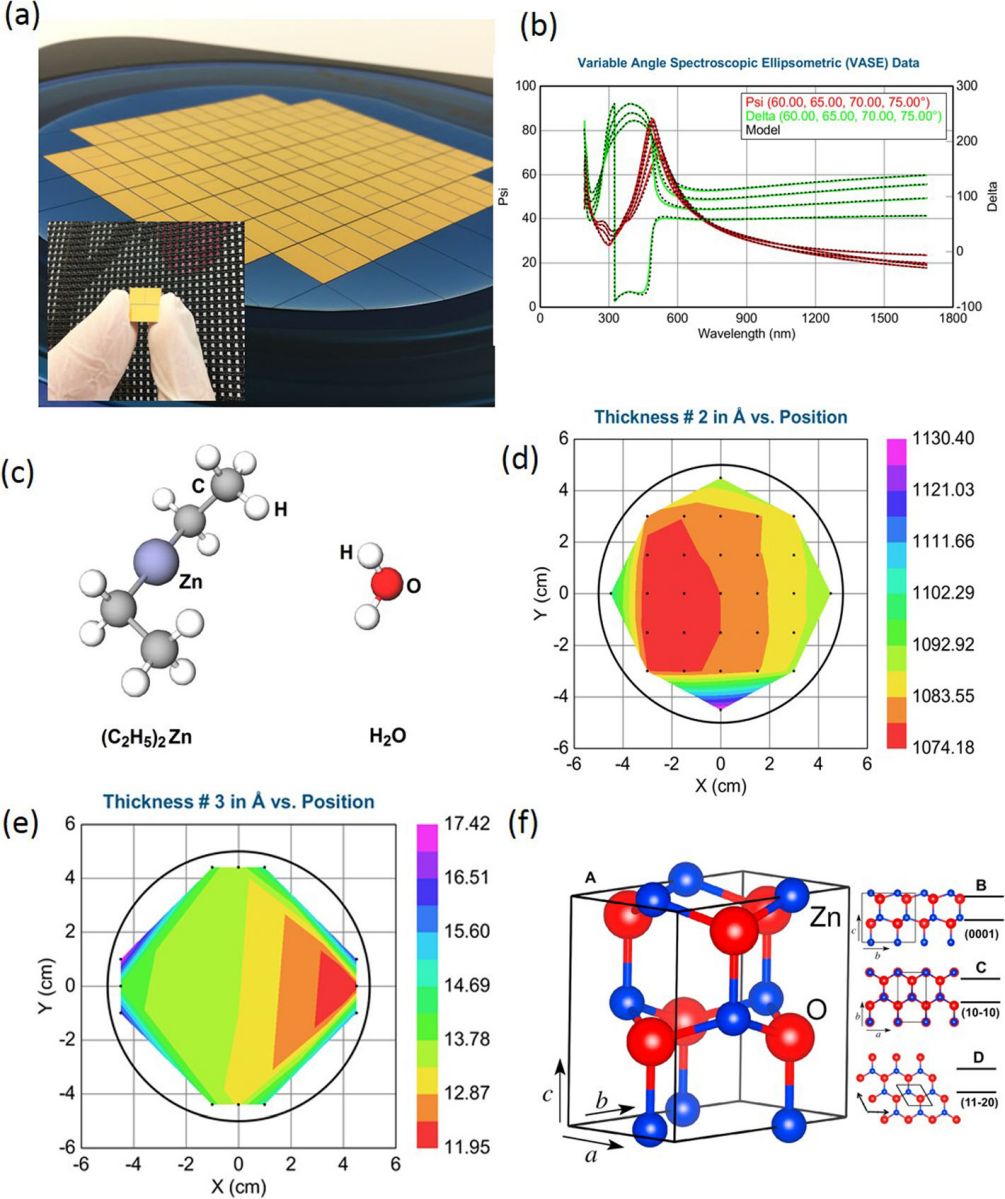
**a** Angular optical image of the wafer-scale ALD deposited ZnO on Au electrodes with insert—an individual 1-cm^2^ diced structure. **b** Experimental and model generated data for variable angle SE measurements of SiO_2_ deposited on Si wafer. **c** Graphical scheme of (C_2_H_5_)_2_Zn and H_2_O precursors, respectively. **d** SE mapping of the SiO_2_ thickness in Å. **e** SE mapping of the thickness of ZnO films in Ångstrom on a 4-in. Si/SiO_2_ wafer. **f** Unit cell of wurtzite ZnO with red—O and blue—ZnO atoms (panel **a**). Atomic arrangement and (0001), (10-10) and (11-20) lattice planes B, C and D, respectively. The outline of the unit cell is shown as a black line. Reprinted from ref. [30] with permission from Elsevier Science

In this work, wafer-scale 2D Au-ZnO heterostructures were developed by the ALD technique using (C_2_H_5_)_2_Zn and H_2_O precursors, as presented in Fig. 1, and the best deposition parameters were established and summarized in Table 1. Both physical and electrochemical properties of fabricated Au-ZnO heterostructures towards amperometric H_2_O_2_ detection at the low and high H_2_O_2_ concentration levels were subsequently investigated. It was confirmed that the ALD-developed Au-ZnO heterostructures demonstrated high sensitivity and selectivity as well as great long-term stability for the wide linear range of H_2_O_2_ concentrations (1.0 μM to 120 mM), which ensured a great potential for their further implementation in practical electrochemical devices.

**Table 1:** Recipe for ALD development of ZnO nanofilms using (C_2_H_5_)_2_Zn and H_2_O precursors

Parameters	Deposition	
	1	2
Inner heater (°C)	250	250
Outer heater (°C)	250	250
Zn precursor heater (°C)	250	250
Isolate pump	✔	✔
Exposure	–	–
Flow (sccm)	30	30
Pulse *H*_2_*O* (Sec)	0.1	0.1
Exposure (sec)	10	10
Initiate pump	✔	✔
Purge *H*_2_*O* (Sec)	15	15
Isolate pump	–	–
Pulse Zn precursor (Sec)	0.06	0.06
Exposure	–	–
Initiate pump	–	–
Purge Zn precursor (Sec)	10	10
Number of cycles	16 (0.9 nm)	23 (1.3 nm)

(✔) Process is carried out; (–) process is exempted

## Methods

### 2D Au-ZnO Heterostructures

Four-inch Si/SiO_2_ wafers (12 Ω/cm) were used as substrates for ALD depositions of ZnO. It should be mentioned that in order to reduce the potential influence of Si wafer on the subsequent electrical measurements, insulating SiO_2_ layer with a thickness of approximately ~ 100 nm was CVD on all wafers. The CVD of SiO_2_ was done prior to ZnO ALD deposition employing Oxford Instruments PLASMALAB 100. After that, gold (Au) electrodes were fabricated on the developed thick SiO_2_ surface by using optical photo-masks and electron beam evaporation (EBE) (Nanochrome II (Intivac)). Au electrodes were grouped into square 3-electrode structures, which was made on the SiO_2_/Si wafer segments as shown in Fig. 1a. The thickness of all Au electrodes was about ~ 150 nm, which was confirmed by appropriate AFM measurements (See Additional file 1: Figure S1 of Supporting Information). Then, thin-film ZnO were deposited on the top of these Au electrodes by ALD. All wafer-scale ZnO depositions were performed on Savannah S100 (Ultratech/Cambridge Nanotech) equipment. The chamber was heated up to 250 °C. The main precursor—solid diethylzinc [(C_2_H_5_)_2_Zn] (99%, Strem Chemicals)—was heated to 250 °C. Then, H_2_O vapor was used as a second precursor. The thickness of both the CVD SiO_2_ and the growing ZnO films was in situ monitored by spectroscopic ellipsometry (SE) (J.A. Woollam M-2000) over the wavelengths of 250–1690 nm. Specifically, Fig. 1a depicts the angular image of 2D ZnO on the specially designed Au electrodes EBE on 4-in. SiO_2_/Si wafer straight after ZnO deposition. Developed 2D ZnO films appeared to be fully transparent. Subsequent to the ZnO deposition, all SiO_2_/Si wafers were diced into the small segments presented as an insert in Fig. 1a for further post-annealing. Both experimental and model-generated data for variable angle SE measurements of CVD deposited SiO_2_ on Si wafer with SE mapping of the thickness of SiO_2_ in Ångstrom are shown in Fig. 1b. The Cauchy model is used for fitting the SE data in order to obtain the optical properties.

Graphical interpretation of both [(C_2_H_5_)_2_Zn] and H_2_O precursors and experimental data for SE SiO_2_ thickness mapping in Ångstrom are depicted in Fig. 1c and d, respectively. Additional experimental and model-generated data for variable angle SE measurements of ZnO films with an initial thickness of 0.9 and 1.3 nm, respectively, are summarized in Additional file 1: Figure S2 of Supporting Information. In order to ensure the consistency of ALD fabricating ZnO films, several trials carried out at the deposition temperature of 250 °C. Uniformly distributed, defects-free, conformal wafer-scaled ZnO films were obtained. For example, Fig. 1e shows the SE measurements for ~ 1.3-nm-thick ZnO film prior to annealing. As clearly presented in this figure the thickness of approximately 1.3 nm of ZnO film was impeccably distributed over 4-in. SiO_2_/Si wafer. Thus, these ZnO films were selected for post-annealing and further electrochemical H_2_O_2_ detection. In such ultra-thin ZnO nanostructures the (10-10), (11-20), and (0001) planes are repeating the unit cell in the appropriate directions. In this case, wurtzite crystal structure of ZnO from orthorhombic unit cell is obtained [47], as schematically presented in Fig. 1 f. Although all of the planes have non-uniform atomic arrangements, the zigzag pattern of the Zn and O sub-lattices overlaps for the (10-10) and (11-20) planes, whereas the sub-lattices along the *c*-direction for the (0001) plane are slightly shifted [47]. Such shift divides the weight center of negative and positive charges, and consequently, enables polarity of the layers. The developed recipe, including the established deposition parameters for ALD fabrication of ZnO films, is summarized in Table 1.

Finally, after ZnO deposition on Au electrodes, the Au-ZnO heterojunctions were made on each segment of the wafer (insert in Fig. 1a) in the heating chamber. For this purpose, all fabricated ZnO films with a thickness of ~ 1.3 nm deposited Au electrodes were heated up to the temperature of 250 °C in air for the development of Au-ZnO heterostructures. The heating rate was 0.5 °C/min. At such annealing temperature and at the absence of any molecular charge compensation mechanism, the polar surfaces of ZnO are unstable and thus undergo surface reconstructions and agglomeration into island-like nanostructures significantly enhancing the *surface-to-volume* ratio of Au-ZnO heterostructures. Then, after annealing, the developed Au-ZnO heterostructures were utilized for further materials characterization experiments. Thus, unless it is stated otherwise, these heterostructures were subsequently used for amperometric H_2_O_2_ detection.

### Characterization

The surface morphology of Au-ZnO heterostructures was investigated by FE-SEM (JEOL 7800F) and by AFM (JPK System, Nano Wizard). XPS measurements were used for establishment the chemical state and surface composition of the developed heterostructures (Rigakudenki model, 7000 with monochromatic Mg-Kα radiation at 300 W). Binding energy of 284.6 eV for C 1 s is utilized for calibration and correction of the obtained binding energy values. In addition, Raman spectroscopy measurements were used to determine the vibrational modes of molecules, identify the crystallinity of developed heterostructures and their structural fingerprint. They were performed on (Lab Ram ARAMIS, Horiba Jobin-Yvon, Edison) Raman spectrometer equipped with λ = 532.2 nm Ar-ion laser. The lattice vibrations of ZnO were investigated and recorded by Fourier transform infrared spectroscopy (FTIR) spectrometer (Shimadzu IR Prestige 21). H_2_O_2_ sensing properties of the developed Au-ZnO heterostructures were electrochemically measured at the room temperature on Autolab PGSTAT204 (Metrohm Autolab, B.V.). For this purpose 3-electrode system is designed. In this system, Pt wire represents the counter electrode, Au-ZnO heterostructure with the area of 0.5 cm^2^ acts as the working electrode, and Ag/AgCl (3.0 M KCl) used as the reference electrode, respectively. The potential range from − 0.9 to 0.2 V was used for current-voltage (CV) measurements at the room temperature of 20 °C. In addition, both chronoamperometry and EIS measurements were used at various conditions for electrochemical behavior evaluation of ALD-fabricated Au-ZnO heterostructures. Specifically, an applied potential of 0.14 V was utilized for chronoamperometry, where the H_2_O_2_ concentration was gradually increased stepwise at the magnetic stirring of 330 rpm. Moreover, EIS experiments were performed in 1 mM K_4_Fe(CN)_6_ solution containing 0.1 M KCl. In these experiments, the frequency changed from 0.1 to 10^5^ Hz.

## Results and discussion

### Characteristics of Au-ZnO heterostructures

**Fig. 2:**
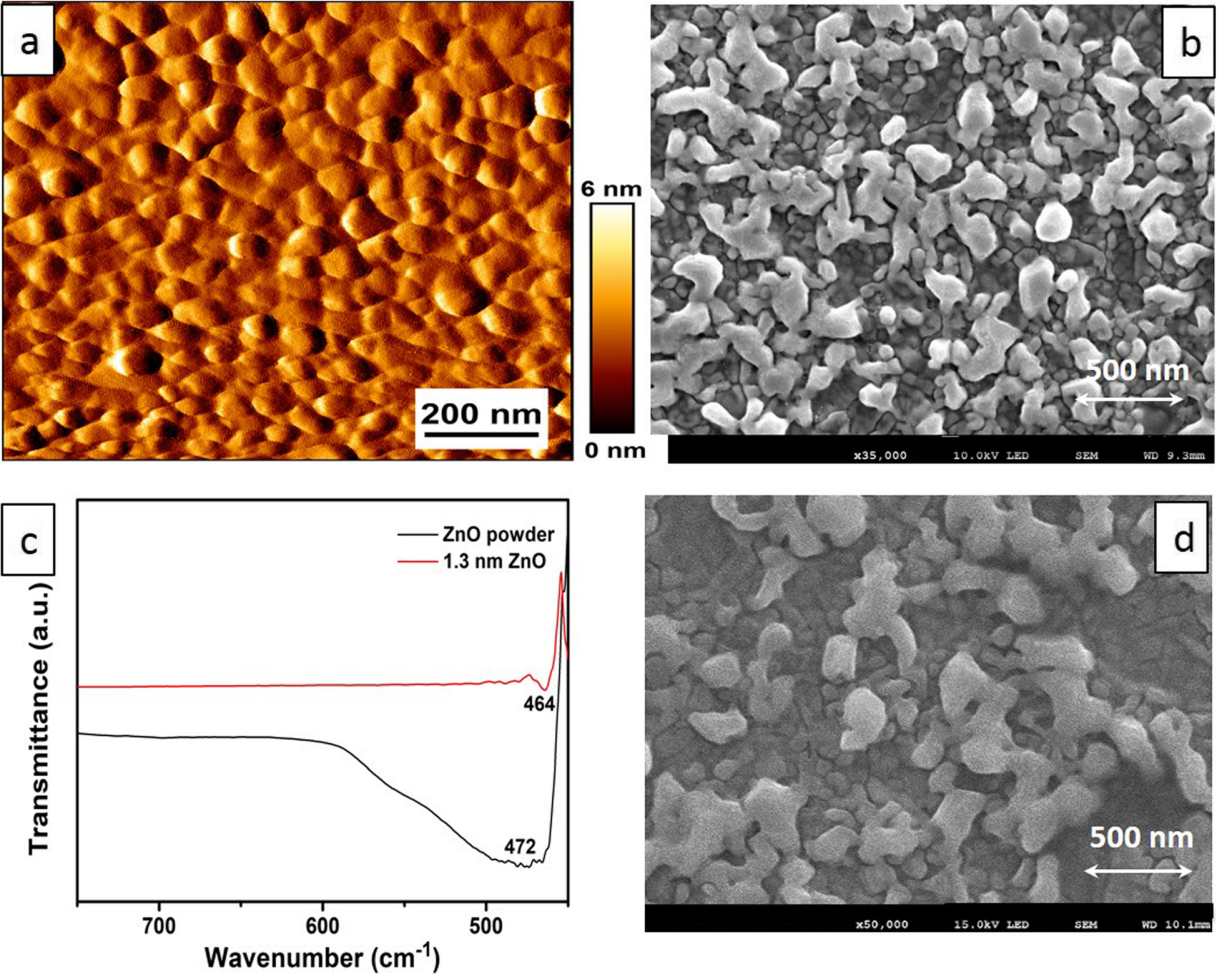
**a** AFM image of Au electrode on Si/SiO_2_ wafer. **b** FE-SEM image of ALD-fabricated Au-ZnO heterostructures after annealing at 250 °C. **c** FTIR spectra of ALD-developed ~ 1.3-nm-thick ZnO on Si/SiO_2_ substrate and commercial ZnO powder. **d** FE-SEM image of the Au-ZnO heterostructures after their long-term stability tests

Figure 2 combines both AFM and FE-SEM images representing the established morphology of Au electrodes and developed Au-ZnO heterostructures, respectively, after post-deposition annealing at 250 °C for 3 h. Specifically, Fig. 2a shows the AFM measurement of the flat Au electrode on Si/SiO_2_ wafer prior to annealing. It was evidently observed in this electrode that the average size of Au nanoparticles was about ~ 50–80 nm and their average height was approximately 3.0 nm. The uniform and smooth distribution of all Au nanoparticles on the surface of the SiO_2_/Si substrate was observed. On the contrary, Fig. 2b shows FE-SEM picture of the surface morphology of Au-ZnO heterostructures after post-annealing at 250 °C. Compared with the similar thickness of annealed ultra-thin WO_3_ [8] and TiO_2_ [12] films deposited by ALD, the FE-SEM image of Au-ZnO heterostructures depicts quite a rough surface. It is clearly visible that the ZnO films due to their extremely thin thickness were broken, aggregated, and finally agglomerated into island-like Au-ZnO heterostructures under the high annealing temperature. Nevertheless, similar results for ALD-fabricated monolayer WO_3_ were reported [8]. The reason for the development of such an island-like structure is the selection of the deposition temperature within the ALD window for (C_2_H_5_)_2_Zn precursor and the annealing time. The deposition temperature of 250 °C for (C_2_H_5_)_2_Zn precursor testifies for the bigger ZnO grain deposition and was consistent with the previous report [46]. Furthermore, the post-annealing alters stoichiometry, charge carriers concentration, and their mobility. It is also enhanced the *surface-to-volume* ratio of the developed heterostructures ensuring their high electrochemical sensitivity towards the measuring chemical [14]. Thus, the overall properties of the nanometer-thick ZnO films deposited on Au electrodes are determined not only by the parameters of the ALD process but also by the post-growth treatment such as thermal annealing. Moreover, the impact of annealing time of post-annealing of ZnO films on their developed properties was properly evaluated [54]. Noteworthy, the size of obtained ZnO nano-grains in the Au-ZnO heterostructures in our investigation was consistent with the published results for the chosen annealing time [55]. Meanwhile, Fig. 2c represents the lattice vibrations in the perturbation area of FTIR measurements for both pure ZnO powder and ALD-fabricated ZnO film with a thickness of 1.3 nm. Peaks of 464 and 472 cm^−1^, representing the Zn-O stretching vibrations for the tetrahedral surrounding of Zn atoms [56], were collected for the 1.3-nm-thick ZnO film and commercial ZnO powder, respectively. As is demonstrated in Fig. 2c, the peak for the ALD-fabricated 1.3-nm-thick ZnO film was slightly shifted compared with the same peak measured for the commercial ZnO powder and was consistent with previous analysis [55]. Considering the possibility that the nanostructured ZnO may not be stable in either acidic or base solutions, additional FE-SEM measurements of Au-ZnO heterostructures after their long-term stability tests (more than 30 consecutive days of testing) were also taken place. The obtained FE-SEM images for Au-ZnO heterostructures (Fig. 2d) have clearly confirmed and testified that the surface morphology of samples used for the long-term stability tests has not been changed.

**Fig. 3:**
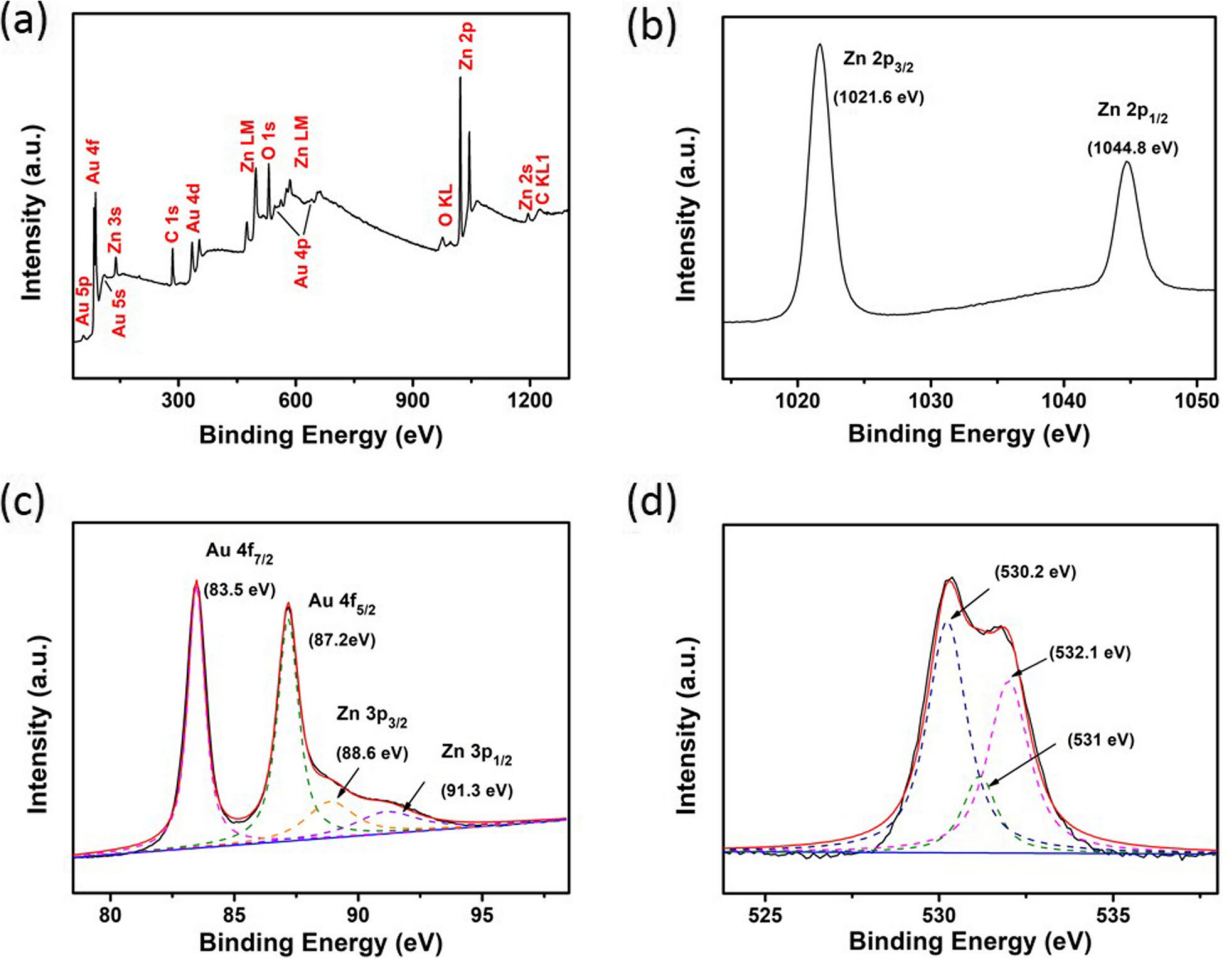
XPS spectra of 2D ZnO-Au heterostructures. **a** Full survey scan spectrum. **b** Zn 2p region. **c** Au 4f and Zn 3p regions. **d** O 1 s region

Unfortunately, the employment of a well-established X-ray diffraction technique for crystallinity characterization of the developed Au-ZnO heterostructures was not possible owing to their extremely thin thickness. Therefore, the surface-sensitive quantitative spectroscopic XPS measurements were performed on such heterostructures to establish the elemental composition at the parts per thousand range and chemical and electronic states of the elements that exist within the heterostructure. A wide-scan survey spectrum of the developed Au-ZnO heterostructures is displayed in Fig. 3a, where the main Au, O, and Zn elements can be clearly identified. A survey spectrum is usually the starting point of XPS analyses because it shows all elements present on the sample surface. The spectrum was calibrated using the C 1 s peak at 284.8 eV. Peaks of Au 4d, Au 4f, and C 1 s are also present in the survey, which reflected from the Au electrode and calibration reference for binding energies of the peaks, respectively. The high-resolution scan of Zn 2p is presented in Fig. 3b. The doublets of Zn 2p_3/2_ and Zn 2p_1/2_ were displayed at 1021.6 and 1044.8 eV, respectively. The binding energy difference of 23.2 eV between these two Zn 2p_3/2_ and Zn 2p_1/2_ peaks confirmed the presence of zinc in Zn^2+^ state [57, 58] within the heterostructure. Moreover, the results obtained are very consistent with the previously reported parameters for ZnO thin films [52]. The high-resolution scan for Au 4f and Zn 3P is depicted in Fig. 3c. Two deconvoluted peaks of Au 4f_7/2_ and Au 4f_5/2_ have a close similarity to the respective binding energies at 83.5 and 87.2 eV in the Au 4f core-level spectrum for the developed Au-ZnO heterostructure. Characteristically, the ~ 3.7-eV separation between the maximum intensity of the peaks at 83.5 and 87.2 eV perceived in the Au-ZnO heterostructure could be ascribed to the spin-orbit components Au 4f_7/2_ and Au 4f_5/2_ of the metallic gold [59–63]. Quite intriguing was the fact that the recorded 3.7-eV peak separation is very similar to the separation of the same peaks in the previous report [63]. Both Au 4f_7/2_ Au 4f_5/2_ peaks exhibited the downward shift in binding energy accompanied by the increase in Au incorporation for Au° and Au^+^ states. In addition, two very weak peaks corresponding to Zn 3p_3/2_ and Zn 3p_1/2_ were also readable at 88.6 and 91.3 eV, respectively.

The O 1 s spectrum of Au-ZnO heterostructures is shown in Fig. 3d. Typically, for O 1 s peaks in heterostructures, the obtained O 1 s peaks for Au-ZnO heterostructure are deconvoluted into 3 major sub-peaks. For instance, the peak at 530.2 eV is assigned to O^2−^ ions of Zn-O bonding reported for the wurtzite ZnO structure [64]. Another peak at 532.1 eV is ascribed to both O^−^ and O^2−^ ions in the oxygen-deficient regions of the ZnO matrix. Remarkably, the intensity of these peaks partially associated with the changes in the oxygen vacancy concentration [65]. In addition, peak at 530.0 eV is a reflection of the adsorption of O_2_, H_2_O, and CO_2_ oxygen-containing molecules on the Au-ZnO heterostructure surface [66]. It should be stressed that the binding energies of the observed 3 sub-peaks are coherent with the previously published Gaussian peaks at ~ 530.2, 531.1, and 532 eV, respectively [65].

**Fig. 4:**
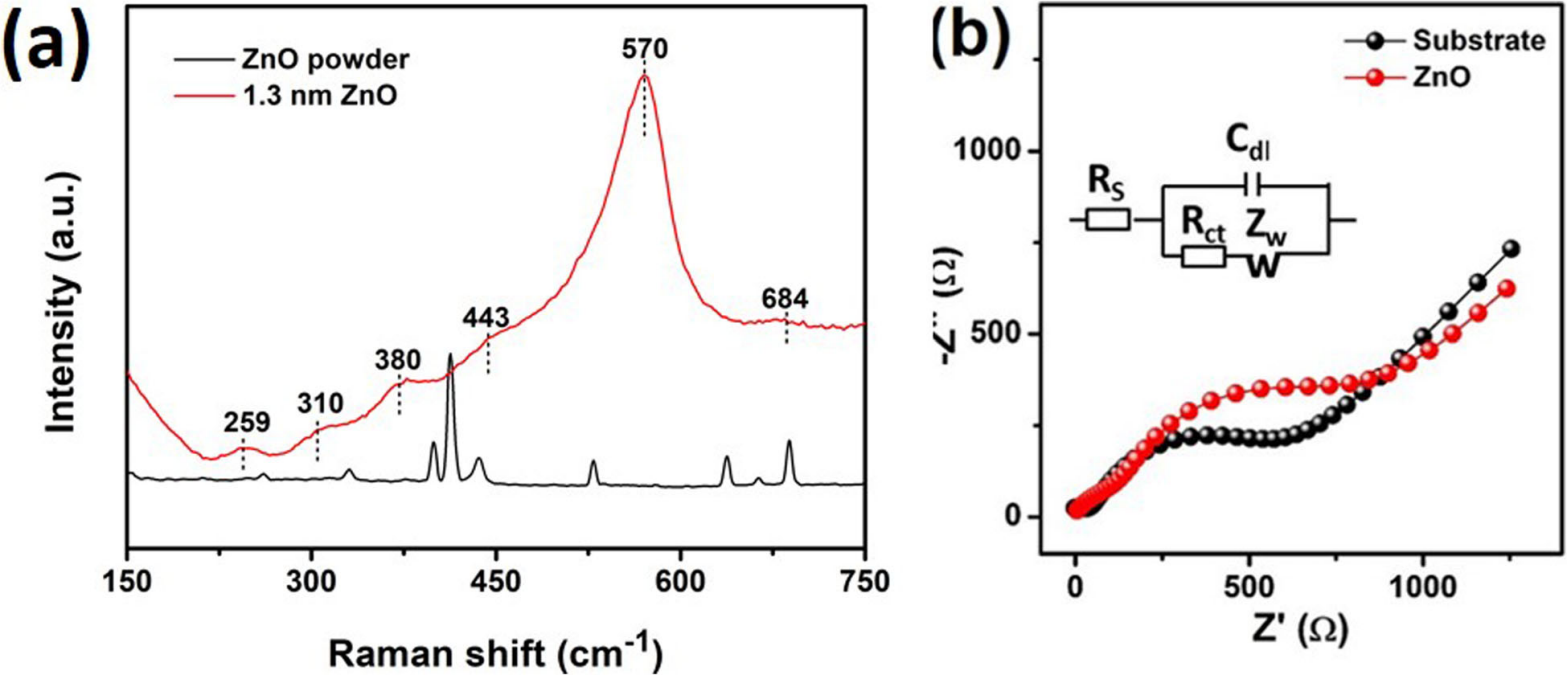
**a** Raman spectra at 25 °C for ZnO powder and ALD-developed ZnO with thickness 1.3 nm. **b** Nyquist plots of the blank substrate and ZnO with 5 mM K_4_Fe(CN)_6_ solution

Raman spectroscopy is a contactless and non-destructive material quality analyzing technique. It was employed for the analysis of the phase orientation, transport, and vibration properties. Thus, the recorded Raman spectrum for ALD-fabricated ZnO film with a thickness of 1.3 nm is presented in Fig. 4a. Equally important, the Raman spectrum for the commercial ZnO powder is also incorporated into Fig. 4a for comparison. The main characteristic helping to identify the wurtzite ZnO phase is a non-polar phonon mode (E_2_^high^) connected to the lattice vibration of the O_2_ atoms observed at ~ 430 cm^−1^. It is interrelated to the lattice vibrations of Zn atoms [66]. Other E_2_^high^ phonon modes for the ZnO powder were noticed at ~ 438.9 and 440.4 cm^−1^, respectively. It is quite prominent that the observed E_2_^high^ phonon mode depicted in Fig. 4a shifted from its standard value (437 cm^−1^), appeared to designate the existence of compressive strain in ZnO powder as well as in ALD fabricated 2D ZnO film. However, compared with the ZnO powder, 3 additional peaks at ~ 259, 570, and 684 cm^−1^ for 2D ZnO film were recorded. Specifically, a high intensity of the peak at 570 cm^−1^ is noticeable, which can be attributed to the high concentration of the developed oxygen vacancies in ZnO nanostructure [67]. Noteworthy, this mode is absent in the spectrum for ZnO powder. However, in our case, the observed intensity of the 570-cm^−1^ peak is too high, which may also be ascribed to the overlapping A_1_(LO) and E_1_(LO) phonon modes. Considering the fact that the surface optical modes are usually observed between the A_1_(TO) and A_1_(LO) polar phonon modes [66], the huge intensity of the peak at 570 cm^−1^ could also be assigned to the intrinsic defects of the host-lattice induced by the development of the heterostructure [68–70]. Nevertheless, our investigation confirmed that the ALD fabricated 2D ZnO films possess high crystalline and optical properties in contrast to the commercial ZnO powder. Furthermore, the influence of the point defects which appeared in the 2D ZnO films on the anomalous intensity of the Raman modes can be disregarded.

Figure 4b expresses plotted impedance spectra of both wafer substrate and Au-ZnO heterostructure. The impedance results for the substrate as well as for the Au-ZnO heterostructure were obtained using the Randles equivalent circuit. The temperature of the buffer 5 mM K_4_Fe(CN)_6_ solution was 25 °C. The large Nyquist plot with *R*_et_ = ~ 1.5 kΩ was recorded for the Au-ZnO heterostructure, while the smaller Nyquist semicircle with *R*_et_ = ~ 0.9 kΩ was measured for the wafer substrate. Although the thickness of the Au-ZnO heterostructure was very thin, its contribution to the overall heterostructural impedance was found to be substantial and noticeable.

### H_2_O_2_ sensing properties

**Fig. 5:**
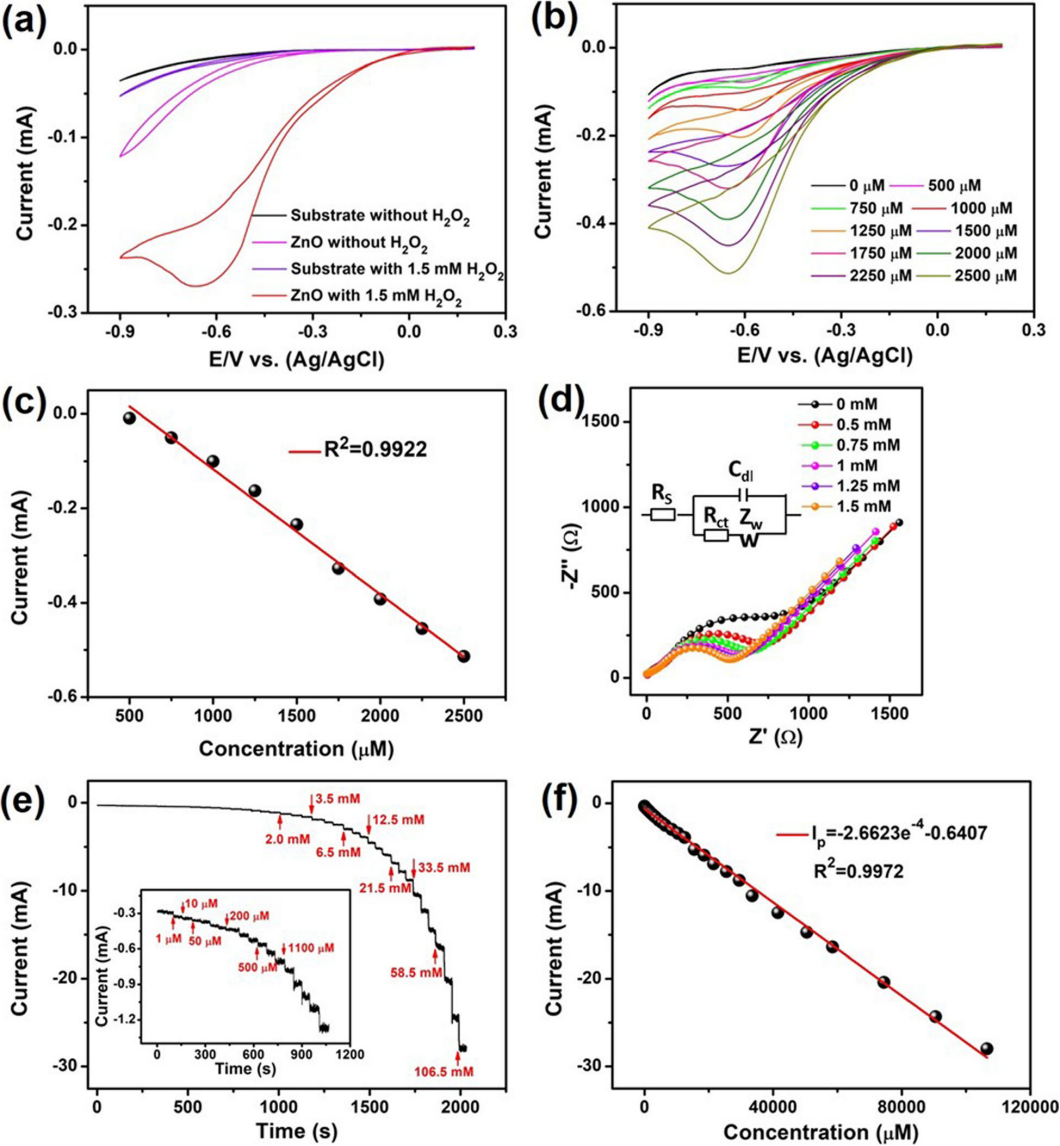
**a** Measured CVs for the blank substrate and 1.3-nm-thick ZnO electrode at the absence and presence of 1.5 mM H_2_O_2_. **b** CV curves of ZnO with the different concentrations of H_2_O_2_. **c** Corresponding linear plot of the current peak versus H_2_O_2_ concentration. **d** Nyquist plots of 1.3-nm-thick ZnO with various H_2_O_2_ concentrations in 5 mM K_4_Fe(CN)_6_ solution. **e** Chronoamperometric current response of the Au-ZnO heterostructure to the changes of H_2_O_2_ concentration. **f** Corresponding linear plot of the current versus H_2_O_2_ concentration

The electrochemical performance of the sensor based on Au-ZnO heterostructures with an area of approximately 1.0 cm^2^ towards H_2_O_2_ was performed using the 3-electrode system, as presented in detail in the “Methods” section. Remarkable improvement towards amperometric H_2_O_2_ detection was clearly observed between the blank substrate and the sensor based on Au-ZnO heterostructure, as shown in Fig. 5a. In order to investigate it further, CV measurements at the different H_2_O_2_ concentrations (Fig. 5b) continued until the upper detection limit of 120 mM was established. Figure 5c displays great linearity between the recorded peak current and the measured H_2_O_2_ concentration, which reaffirmed the effective electro-catalytic activity of Au-ZnO heterostructures. A fast response-recovery time of approximately ~ 2.0 s for the amperometric H_2_O_2_ detection was obtained. This response-recovery time indirectly resonated rapid electron transfer reactions at the Au-ZnO heterostructures. It could be also due to the high concentration of the oxygen vacancies on the heterostructure surface. Noteworthy, the EIS measurements, presented in Fig. 5d, showed that as the H_2_O_2_ concentration in the K_4_Fe(CN)_6_ solution increased, the measured Nyquist plot is gradually getting to be smaller and the R_et_ value proportionally decreased, which indicates the improved electron transfer rate. It should be stressed that the low R_et_ is an indirect confirmation of the presence of diffusion-limited process between the Au-ZnO heterostructure surface and the 5 mM K_4_Fe(CN)_6_ solution [71]. The correlated linear regression equation could be presented as follows: *E*_P_ (V) = 0.0671 log_ν_ + 0.278. For the diffusion-controlled process mentioned above, *E*_P_ can be defined by the following Tafel equation [9]:

1 $$ {E}_P=\left[2.303 RT/2\;\left(1-\alpha \right)\;{n}_{\alpha }F\right]\; lo{g}_v+K, $$

where *R* is the universal gas constant , *T* is the temperature of the solution measured in K, *F* is the Faraday constant, *α* is the electron transfer coefficient, *n*_*α*_ is the number of electron transfers involved in the rate-determining step, and *K* is a constant.

Moreover, chronoamperometry as a time-dependent technique is utilized for the determination of the following parameters of amperometric sensors based on Au-ZnO heterostructures: sensitivity, linear response range and the low limit of detection (LOD) for H_2_O_2_. In this technique, a square-wave potential is applied to the working electrode. The electrode current, measured as a function of time, fluctuates according to the diffusion of H_2_O_2_ from the bulk solution towards the Au-ZnO heterostructure surface. As the current is integrated over the relatively longer time intervals, chronoamperometry provides a better *signal-to-noise* ratio compared with other amperometric techniques. Therefore, chronoamperometric measurements were performed for Au-ZnO heterostructures and the main results are summarized and presented in Fig. 5e. During these measurements, the H_2_O_2_ was added approximately every 50 s. It is clear from Fig. 5e that the sensor based on Au-ZnO heterostructures demonstrated typical current time dynamic responses at changes of concentration from 2.0 μm to 106.5 mM. Responses to much lower H_2_O_2_ concentrations from ~ 1.0 to 1100 μM are shown as insert to Fig. 5e. The response time to the various H_2_O_2_ concentrations was found from *i*–*t* curves. In fact, the Au-ZnO heterostructures have clearly demonstrated the fastest response-recovery time (~ 2.0 s) among all reported ZnO-based electrochemical H_2_O_2_ sensors [28–35]. Corresponding calibration curve for H_2_O_2_ detection by 2D Au-ZnO heterostructures is presented in Fig. 5 f. Noteworthy, the sensor based on Au-ZnO heterostructures signified the remarkable linear dependence of chronoamperometric responses within the wide H_2_O_2_ concentrations range of ~ 1.0 μm–120 mM. It must be stressed that such a wide linear range for the sensor based on ALD-fabricated Au-ZnO heterostructure is the widest linear range among all electrochemical [28–35], chemiluminescent [36, 37, 72], colorimetric [23–25], and fluorometric [37–40] H_2_O_2_ detectors reported to date. In addition to the widest linear measured H_2_O_2_ concentrations range and extremely fast response-recovery time, the low LOD of 0.78 mM was achieved for the Au-ZnO heterostructures. This low LOD value is compatible with the best LOD values reported for colorimetric [23–25] and fluorometric [39] H_2_O_2_ sensors and is better than the reported LOD value for chemiluminescent [36, 37] H_2_O_2_ detectors. Therefore, the experimental results obtained for Au-ZnO heterostructures have unambitiously verified that these ALD-fabricated heterostructures possess excellent sensing capabilities towards amperometric measurements of the lower H_2_O_2_ concentrations compared not only to the ZnO-based nanostructures but also to the best other H_2_O_2_ detection methods. All characteristics for 2D nano-films and heterostructures reported so far for electrochemical, chemiluminescent, colorimetric, and fluorometric H_2_O_2_ detectors are summarized into Table 2.

**Table 2:** Comparison of characteristics of the H_2_O_2_ sensor based on ALD-developed Au-ZnO heterostructures with H_2_O_2_ sensors based on other nanomaterials and detection modes reported to date

Electrode material	Sensitivity (μA·μM^−1^·cm^−2^)	Linear range (μM)	LOD (μM)	Response time (s)	Refs
Au-ZnO heterostructures	0.53	1.0–120000	0.78	~ 2.0	This work
ZnO thin film	–	10–110	–	~ 115	[35]
Co-doped ZnO nanoparticles	92.44	5000–30000	14.3	–	[28]
Pt-ZnO nanotubes	–	20–5000	1.5	~ 10	[29]
Cyt.c-ZnO nanosheets	2.0	10–1000	0.8	–	[30]
Zno-Co_3_O_4_-NiCo_2_O_4_-Ni foam	388	0.2–2400	0.1	20	[31]
AuNPs-ZnO-NTs	1.34	1.0–3000	0.1	~ 15	[32]
Ag-ZnO nanoflower	50.8	1.0–20	2.5	–	[33]
AgNPs-ZnO	1.64	2–5500	0.42	–	[34]
Au NPs-ZnO NRs	0.15	8–983	0.9	–	[45]
TRP (chemiluminescent)	–	2.0–1000	2.0	–	[36]
KIO_4_/CO_3_^2−^ (chemiluminescent)	–	2.7–600	2.7	–	[37]
C-dots/Fe^2+^/VB_1_ (fluorometric)	–	0.5–450	0.074	–	[39]
Au@Ag NPs/C-dots (fluorometric)		0.50–400	0.20	–	[40]
GQDs/CuO (colorimetry)	–	0.5–10	0.17	–	[23]
Fe_3_O_4_ MNPs (colorimetry)	–	0.50–150	0.25	–	[24]
HPPtCuDs (colorimetry)	–	0.3–325	0.1	–	[25]

**Fig. 6:**
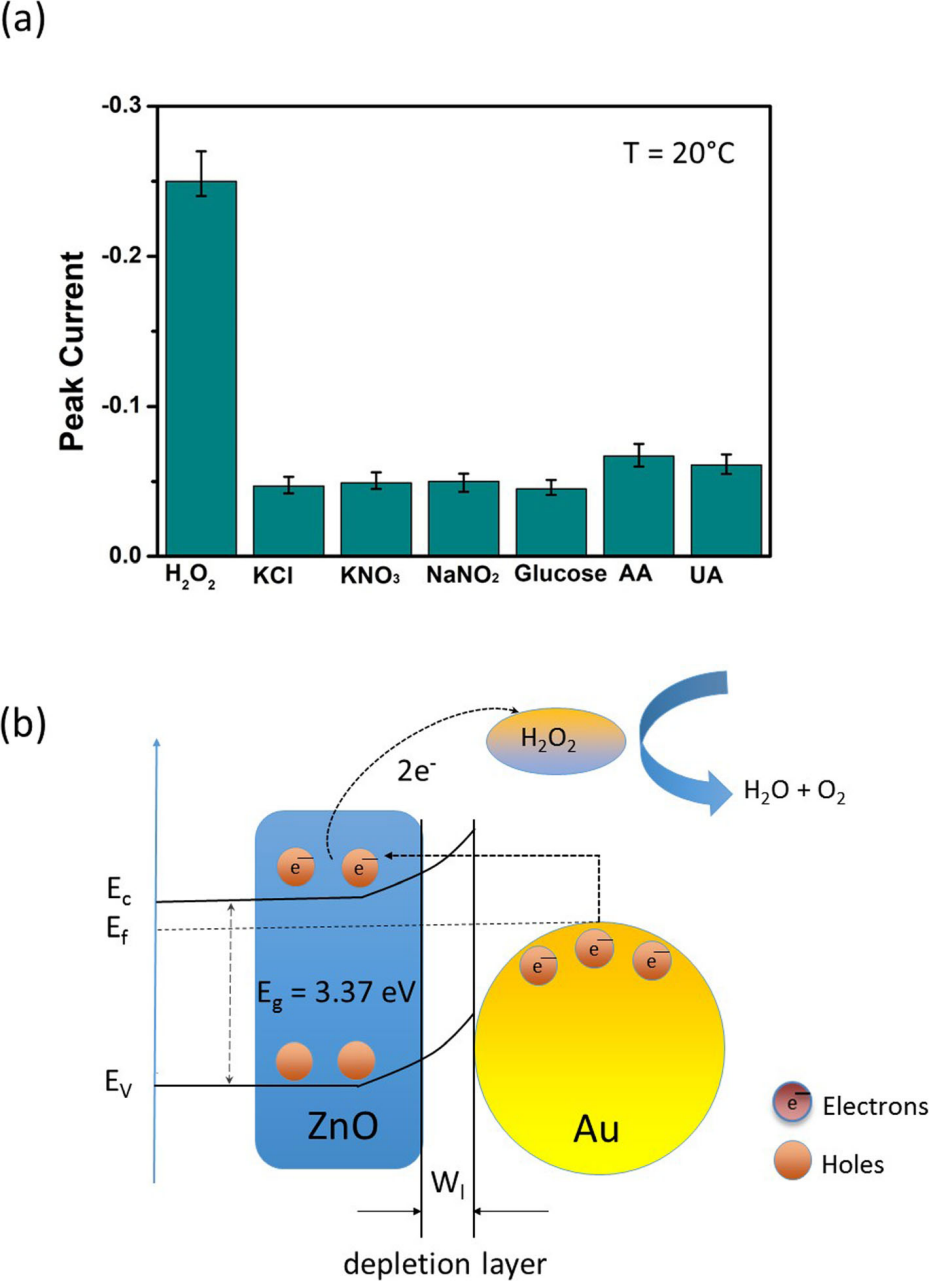
**a** Interference study of the H_2_O_2_ sensor based on Au-ZnO heterojunction at the presence of 10 μM H_2_O_2_ and 1.0 mM of different interfering chemicals at 25 °C. **b** Schematic band diagram of Au-ZnO heterostructure under exposure to H_2_O_2_

Besides the widest linear H_2_O_2_ concentration range, low LOD, and the fast response-recovery time, the cross-reference of Au-ZnO heterostructures to the other chemical agents existing in the measuring solution has also been evaluated at room temperature. For this purpose, 1.0 mM of the different chemicals were subsequently introduced into the measuring solution containing 10 μM H_2_O_2_. Responses of 2D Au-ZnO-heterostructures to the different interfering agents including glucose, KCl, NaNO_2_, AA, UA, and KNO_3_ are displayed in Fig. 6a. It should be stressed that the concentration of the additional chemicals was about 100 times higher (1000 μM) and about 50 times higher for glucose (500 μM) than the existing H_2_O_2_ concentration (10 μM) in the solution. Each interfering chemical agent was tested at least 10 times and the data in Fig. 6a represent the average values obtained after 1000 s of measurement. It is therefore evident from this figure that the 2D Au-ZnO heterostructures were almost insensitive to all added chemicals confirming superior selectivity towards the amperometric H_2_O_2_ detection at room temperature.

Finally, the long-term stability testing is organized and executed for 30 consecutive days with periodical testing every 6 days. The measured results of this testing exhibited that the sensor’s response during the trial has only decreased ~ 3% after 30 days of continuous measurements. This fact reassured that in addition to the main investigated sensing characteristics, the ALD-fabricated nanoscale Au-ZnO heterostructure possesses great long-term stability towards H_2_O_2_ amperometric measurements.

The enhanced mechanism of the amperometric H_2_O_2_ detection for the Au-ZnO heterostructures is presented as the schematic band diagram in Fig. 6b. The improvement in H_2_O_2_ detection by the Au-ZnO heterostructures can be explained by the following reasons. First, the Au-ZnO heterostructures possess a unique tortuous nanostructure ensuring high surface-to-volume ratio and abundance of the adsorption centers on the surface and in the bulk of a heterostructure. Secondly, a tortuous Au-ZnO nanostructure provides enhanced specific surface area as well as big pore sizes with diffusion channels to the measuring chemical, so that the inner surface of the heterostructure would also able to have sufficient contact with H_2_O_2_. Thirdly, junctions between these small nanoparticles in the heterostructure are indistinguishable to the thin film grain boundaries. Thus, the adsorption of reactive O^−^ oxygen ions enabled by high concentrations of the oxygen vacancies developed in the Au-ZnO heterostructure [73, 74]. Fourth, the improved H_2_O_2_ sensitivity is also implemented by the establishment of a well-developed Au-ZnO interface during post-annealing. In regard to electronic sensitization effect, for ZnO, as a typical *n*-type semiconductor, its work function of 4.65 eV is smaller than the work function of Au (5.1 eV) [75]. Consequently, the electrons tend to transfer from a high energy state (ZnO) to a low energy state (Au). Therefore, as for the Au-ZnO heterostructure, a Schottky contact between Au and ZnO would form at the thermal equilibrium state. Accordingly, the Schottky contacted between Au and ZnO results in the generation of a thick depletion layer at the Au-ZnO interface as presented in Fig. 6b. The depletion layer on the Au-ZnO interface was a thick space charge layer in contrast to the bare ZnO-based sensors, which only had a thin surface depletion layer [76]. This not only causes an increase in the resonant electron density but also creates energetic electrons in the higher energy states. As a result, these resonant electrons are so active that they can transfer back to the conduction band of ZnO upon its exposure to the detecting H_2_O_2_ chemical, which affects the reduction of the thickness of the electron depletion layer. Moreover, during oxidation of H_2_O_2_, the presence of Au in the Au-ZnO heterostructure reduces the Schottky barrier, which subsequently ensures an easier and faster way for the sensing reaction [77].

All major characteristics of the various electrochemical, colorimetric, fluorometric, and chemiluminescent H_2_O_2_ detectors are summarized in Table 2. The results obtained for ALD-fabricated Au-ZnO heterostructures can clearly confirm that the 2D Au-ZnO heterostructures have superior performance compared with the other modified ZnO-based nanostructures. Specifically, results showed that our sensors have the widest linear range and the lowest LOD value compatible with the best LOD values among all reported electrochemical, chemiluminescent, colorimetric, and fluorometric H_2_O_2_ detectors. Therefore, Au-ZnO heterostructures are very attractive and reliable candidates for the further development of practical H_2_O_2_ measuring devices.

## Conclusions

In this study, wafer-scale nanometer-thick 2D ZnO films were successfully fabricated on the nanostructured Au electrodes by the ALD technique using (C_2_H_5_)_2_Zn and H_2_O precursors, respectively. Then, for the development of Au-ZnO heterostructures, 2D ZnO films were subjected to post-annealing at 250 °C for 3 h. After annealing, the H_2_O_2_-sensing capabilities of Au-ZnO heterostructures were subsequently investigated. Intensive testing revealed that Au-ZnO heterostructures showed excellent sensing capabilities towards amperometric H_2_O_2_ detection, principally at the low concentration level. 2D Au-ZnO heterostructures displayed a high sensitivity of 0.53 μA μM^−1^ cm^−2^, the widest linear H_2_O_2_ concentration range of 1.0 μM–120 mM among all reported different H_2_O_2_ sensors, very low LOD of 0.78 μM, fast response-recovery time (~ 2.0 s), excellent selectivity in the presence of various interfering chemical agents, and outstanding long-term stability. Additionally, the development of Au-ZnO nano-interface and high *surface-to-volume* ratio in heterostructures enabled highly expanded specific surface area of heterostructure, allowing the measuring chemical agent to reach inside of the heterostructure more easily. Consequently, the study of 2D Au-ZnO heterostructures indisputably validated the fact that these heterostructures can be considered as one of the valuable candidates for the design of practical electrochemical H_2_O_2_ devices.

## Supplementary information

**Additional file 1: Figure S1.** Ellipsometry data for deposition of thicker ZnO for building reliable optical constants (a), device schematic indicating Au/Cr pads in dark green (separated by 200 μm) and the dicing/alignment in markers in light green (20 μm) (b), microcrograph of the edge of four individual devices, as per the mask design a 40 μm gap is allotted for dicing and each Au/Cr pad is inset from the edge of the 1.0 x 1.0 cm die by 50 μm (c), micrograph of 200 m Au/Cr pad separation of 2 individual devices (d) with 3D AFM image of Au/Cr layer step height (e). **Figure S2.** Experimental and model generated data for variable angle spectroscopic ellipsometric measurements of ALD developed ZnO with initial thickness of (a) 0.9 nm and (b) 1.3 nm.

## Data Availability

Surface morphology of the samples was investigated by FE-SEM (Fig. 2). The chemical bonding and crystal structure of the ALD-fabricated samples were characterized by FTIR (Fig. 2), XPS (Fig. 3), Raman spectroscopy (Fig. 4), electrochemical impedance spectroscopy (Fig. 4), and CV measurements (Figs. 5 and 6).

## Abbreviations

AFM: Atomic force microscopy
ALD: Atomic layer deposition
CV: Current voltage
CVD: Chemical vapor deposition
EDS: Energy dispersive spectroscopy
FE-SEM: Field emission scanning electron microscopy
FTIR: Fourier Transform Infrared
ISE: Electrochemical impedance spectroscopy
SEM: Scanning electron microscopy
XPS: X-ray photoelectron spectroscopy
