# Supplementary material for: Nanoscale Au-ZnO Heterostructure Developed by Atomic Layer Deposition Towards Amperometric H2O2 Detection
Source: Nanoscale Res Lett. 2020 Feb 17;15:41. doi: 10.1186/s11671-020-3273-7 (PMC7026348; doi:10.1186/s11671-020-3273-7)
Supplement: Supplementary file 1 — Additional file 1: Figure S1. Ellipsometry data for deposition of thicker ZnO for building reliable optical constants (a), device schematic indicating Au/Cr pads in dark green (separated by 200 μm) and the dicing/alignment in markers in light green (20 μm) (b), microcrograph of the edge of four individual devices, as per the mask design a 40 μm gap is allotted for dicing and each Au/Cr pad is inset from the edge of the 1.0 x 1.0 cm die by 50 μm (c), micrograph of 200 m Au/Cr pad separation of 2 individual devices (d) with 3D AFM image of Au/Cr layer step height (e). Figure S2. Experimental and model generated data for variable angle spectroscopic ellipsometric measurements of ALD developed ZnO with initial thickness of (a) 0.9 nm and (b) 1.3 nm. [file 11671_2020_3273_MOESM1_ESM.docx]

**Supporting Information**

**Nanoscale Au-ZnO heterostructure developed by atomic layer deposition toward amperometric H_2_O_2_ detection**

Hongyan Xu^a+^ , Zihan Wei^b,c+^, Francis Verpoort^b,c,d^, Jie Hu^e^, Serge Zhuiykov^*a,b^

^a^ School of Materials Science and Engineering, North University of China, Taiyuan 030051, PR China

^b^ Ghent University Global Campus, Department of Green Chemistry & Technology, 119 Songdomunhwa-ro, Yeonsu-gu, Incheon 21985, South Korea

^c^ Faculty of Bioscience Engineering, Ghent University, Coupure Links 653, Ghent 9000, Belgium

^d^ State Key Laboratory of Advanced Technology for Materials Synthesis and Processing, Center for Chemical and Material Engineering, Wuhan University of Technology, Wuhan, PR China

**^e^** College of Information Engineering, Taiyuan University of Technology, Taiyuan, 030024, Shanxi, PR China


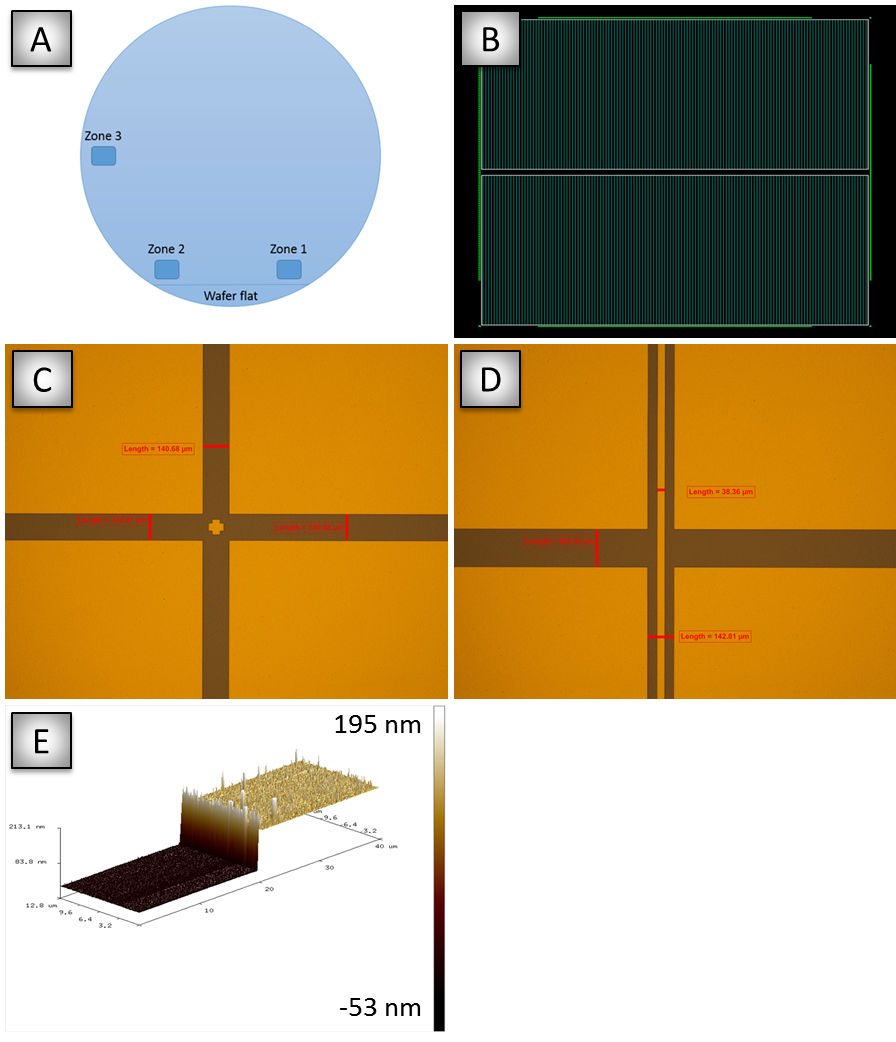


**Figure S1.** Ellipsometry data for deposition of thicker ZnO for building reliable optical constants (a), device schematic indicating Au/Cr pads in dark green (separated by 200 µm) and the dicing/alignment in markers in light green (20 µm) (b), microcrograph of the edge of four individual devices, as per the mask design a 40 µm gap is allotted for dicing and each Au/Cr pad is inset from the edge of the 1.0 x 1.0 cm die by 50 µm (c), micrograph of 200 m Au/Cr pad separation of 2 individual devices (d) with 3D AFM image of Au/Cr layer step height (e).


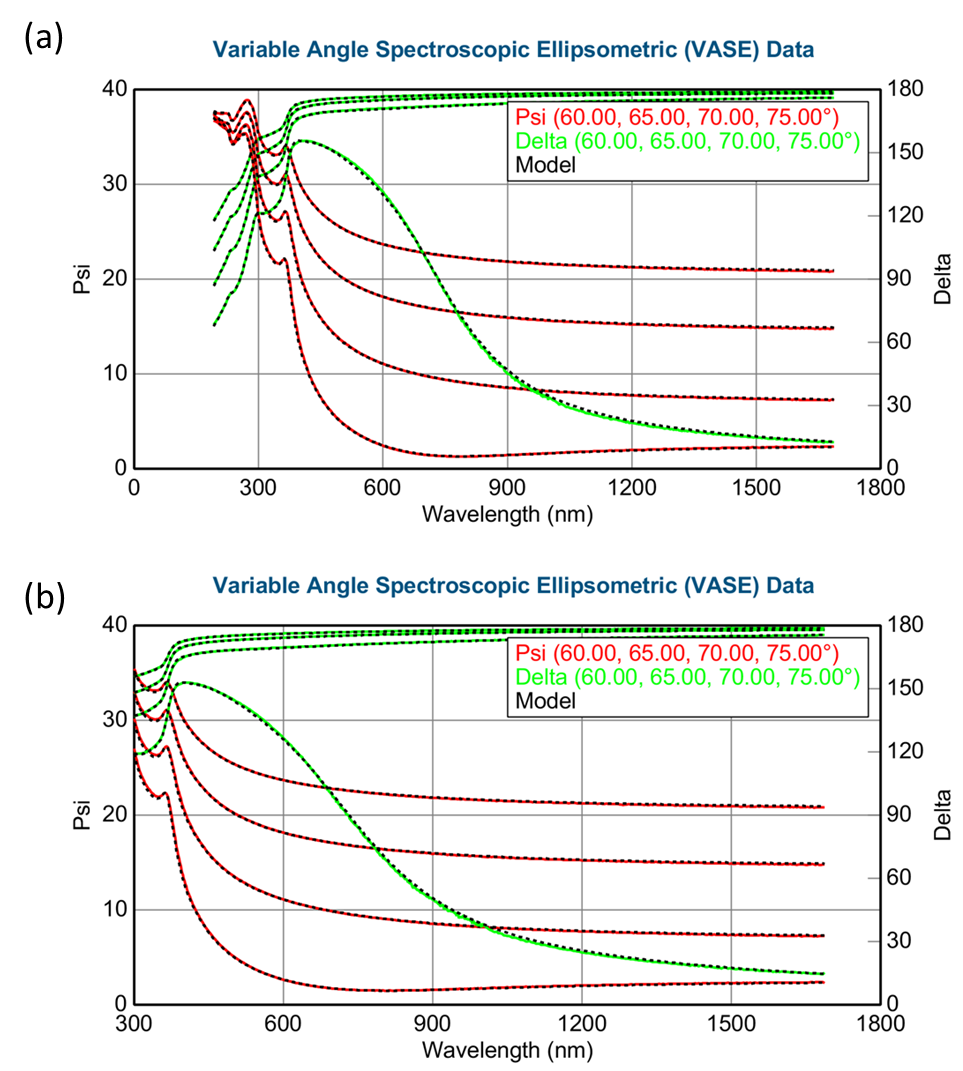


**Figure S2.** Experimental and model generated data for variable angle spectroscopic ellipsometric measurements of ALD developed ZnO with initial thickness of (a) 0.9 nm and (b) 1.3 nm.
